# Effects of dietary camelina, flaxseed, and canola oil supplementation on inflammatory and oxidative markers, transepidermal water loss, and coat quality in healthy adult dogs

**DOI:** 10.3389/fvets.2023.1085890

**Published:** 2023-03-09

**Authors:** Taylor L. Richards, Scarlett Burron, David W. L. Ma, Wendy Pearson, Luciano Trevizan, Debbie Minikhiem, Caitlin Grant, Keely Patterson, Anna K. Shoveller

**Affiliations:** ^1^Department of Animal Biosciences, University of Guelph, Guelph, ON, Canada; ^2^Department of Human Health and Nutritional Sciences, University of Guelph, Guelph, ON, Canada; ^3^Department of Animal Science, Universidade Federal do Rio Grande do Sul, Porto Alegre, Rio Grande do Sul, Brazil; ^4^Consultant, Spring Hill, TN, United States; ^5^Department of Clinical Studies, Ontario Veterinary College, University of Guelph, Guelph, ON, Canada

**Keywords:** omega-3, omega-6, canine nutrition, skin and coat health, flaxseed oil, canola oil, camelina oil

## Abstract

**Introduction:**

Camelina oil contains a greater concentration of omega-3 (*n*-3) a-linolenic acid (C18:3n-3; ALA) than omega-6 (*n*-6) linoleic acid (C18:2n-6; LA), in comparison to alternative fat sources commonly used to formulate canine diets. Omega-3 FAs are frequently used to support canine skin and coat health claims and reduce inflammation and oxidative stress; however, there is a lack of research investigating camelina oil supplementation and its effects on these applications in dogs. The objective of this study was to evaluate the effects of camelina oil supplementation on coat quality, skin barrier function, and circulating inflammatory and oxidative marker concentrations.

**Methods:**

Thirty healthy [17 females; 13 males; 7.2 ± 3.1 years old; 27.4 ± 14.0 kg body weight (BW)] privately-owned dogs of various breeds were used. After a 4-week wash-in period consuming sunflower oil (*n*6:*n*3 = 1:0) and a commercial kibble, dogs were blocked by age, breed, and size, and randomly assigned to one of three treatment oils: camelina (*n*6:*n*3 = 1:1.18), canola (*n*6:*n*3 = 1:0.59), flaxseed (*n*6:*n*3 = 1:4.19) (inclusion level: 8.2 g oil/100 g of total food intake) in a randomized complete block design. Transepidermal water loss (TEWL) was measured using a VapoMeter on the pinna, paw pad, and inner leg. Fasted blood samples were collected to measure serum inflammatory and oxidative marker concentrations using enzyme-linked immunosorbent assay (ELISA) kits and spectrophotometric assays. A 5-point-Likert scale was used to assess coat characteristics. All data were collected on weeks 0, 2, 4, 10, and 16 and analyzed using PROC GLIMMIX in SAS.

**Results:**

No significant changes occurred in TEWL, or inflammatory and oxidative marker concentrations among treatments, across weeks, or for treatment by week interactions. Softness, shine, softness uniformity, color intensity, and follicle density of the coat increased from baseline in all treatment groups (*P* < 0.05).

**Discussion:**

Outcomes did not differ (*P* > 0.05) among treatment groups over 16-weeks, indicating that camelina oil is comparable to existing plant-based canine oil supplements, flaxseed, and canola, at supporting skin and coat health and inflammation in dogs. Future research employing an immune or exercise challenge is warranted, as the dogs in this study were not subjected to either.

## Introduction

Dogs are unable to produce the omega-6 (*n*-6) linoleic acid (C18:2n-6; LA) and the omega-3 (*n*-3) α-linolenic acid (C18:3n-3; ALA), endogenously, and as such, these must be obtained in the diet (1). Omega-3 fatty acids (FAs) in particular have been linked to numerous health benefits, including a reduction in inflammation and oxidative stress, and improved skin and coat health properties, which are directly associated (2–7).

There is a competitive relationship between the *n*-6 and *n*-3 FA pathways for the use of the Δ5- and Δ6-desaturase and elongase enzymes needed to convert LA and ALA into longer chain FAs. Consequently, a balanced dietary *n*-6:*n*-3 ratio is needed to ensure sufficient conversion to longer chain FAs in both pathways. Specifically, and most notably, LA is converted into arachidonic acid (AA), and ALA is converted into eicosapentaenoic acid (EPA) and docosahexaenoic acid (DHA) (8). Both AA and EPA and DHA are parent compounds for the production of pro- and anti-inflammatory eicosanoids, respectively. An increase in endogenous *n*-6 AA results in a prothrombotic, pro-constructive, and pro-inflammatory state, whereas increased EPA and DHA give rise to resolvins, which are anti-inflammatory and pro-resolving. Greater concentrations of *n*-6 FAs and a higher *n*-6:*n*-3 ratio allow for greater conversion of *n*-6 FAs to AA and more pro-inflammatory effects. In contrast, greater concentrations of *n*-3 FAs and a lower *n*-6:*n*-3 ratio allow for increased production of EPA and more anti-inflammatory effects (9). As a result, excessive amounts of *n*-6 FAs and a high *n*-6:*n*-3 ratio promote the pathogenesis of many inflammatory, autoimmune, and dermatological disorders, whereas greater concentrations of *n*-3 FAs and a low *n*-6:*n*-3 ratio exert suppressive effects (10).

In order to formulate canine diets to meet the ideal *n*-6:*n*-3 ratio of between 5:1 and 10:1, *n*-3 rich ingredients are typically required (11). Two oils commonly used to increase *n*-3 inclusion in canine diets are fish oil, as a result of its high levels of EPA and DHA (180 mg EPA, 120 mg DHA/1,000 mg of oil provided in the most common fish oil capsules in the United States today, however, doses vary widely between supplements), and flaxseed oil, due to its favorable *n*-6:*n*-3 ratio of 1:4.19 (12–15). However, large-scale fish oil production required to meet the demands of the growing pet food industry is not environmentally sustainable long-term, and the high abundance of ALA in flaxseed oil makes it susceptible to oxidation, making its use in commercial diets difficult (12, 15). Additionally, flaxseed crops are sensitive to various climates, diseases, and pests, making both of these options less than desirable (12, 14, 15). Alternative animal-based (beef, 1:0.05; milk, 1:0.07; eggs, 1:0.05) and plant-based (canola, 1:0.59; corn, 1:0.01; soybean, 1:0.12; and sunflower oil, 1:0.00) lipid sources commonly used in canine diet formulations all have higher concentrations of *n*-6 FAs rather than *n*-3 FAs (15–17). This leaves room in the market for an alternative plant-based oil source that is economically and environmentally sustainable, with good shelf-stability and a favorable concentration of *n*-3 FAs that could contribute to achieving the ideal *n*-6:*n*-3 ratio in canine diets.

The oil seed camelina (*Camelina sativa*) is considered a low-input, high-yield crop due to its short growing season and resistance to various seasons, climates, and soil types (18–21). The product of this robust crop, camelina oil, provides a rich source of *n*-3 FAs as a result of its desirable *n*-6:*n*-3 ratio of 1:1.8 (22). Additionally, camelina oil contains high concentrations of tocopherols and polyphenols, which have been associated with improved skin and coat health due to their antioxidant properties (22). Due to camelina oil being naturally high antioxidants as well as having a slightly lower concentrations of *n*-3 FAs in contrast to flaxseed oil, it's shelf-stability is better by comparison (23).

Additional data from this study suggests camelina oil to be safe for canine consumption (24). The inclusion of oil supplements in canine diets is often associated with claims of maintenance or support of skin and coat health, but currently there is no data directly comparing the effects of camelina oil supplementation to the effects of other oils approved for use in pet foods on markers of skin and coat health and inflammation. The objective of this study was to compare the effects of dietary camelina oil supplementation to those of flaxseed oil and canola oil supplementation on skin and coat health and inflammatory and oxidative markers in healthy, adult dogs. Outcomes include changes in oxidative and inflammatory biomarkers and coat quality. Additionally, skin barrier function and integrity was assessed by measuring transepidermal water loss (TEWL). Authors hypothesize that camelina oil (*n*-3:*n*-6 = 1:1.8) is comparable, flaxseed (*n*-3:*n*-6 = 1:4.19) and canola oil (*n*-3:*n*-6 = 1:0.59) in terms of its effects on oxidative and inflammatory markers, coat quality, and TEWL.

## Materials and methods

### Animals and housing

This experiment was approved by the University of Guelph's Animal Care Committee (AUP #4365) and was carried out in accordance with national and institutional guidelines for the care and use of animals. Thirty client-owned, adult (7.2 ± 3.1 years) dogs of mixed sex (17 females: 16 spayed, one intact; 13 males: 10 neutered, three intact), weight (27.4 ± 14.0 kg) and breed participated in this study (Table 1). All dogs were deemed healthy based on their previous medical history as well as a pre-study physical examination performed by a licensed veterinarian, complete blood count (CBC), and serum biochemistry profile. During the recruitment process, dogs were excluded if they had any skin conditions, received any pro- or anti-inflammatory medications 2-months prior to baseline samples, had abnormalities on their physical examination, CBC, or serum biochemistry, or were younger than 2 years of age. Dogs were housed at their owners' homes for the duration of the study, they followed their usual daily routines. Pet owners were instructed to provide no supplements, medications, antibiotics, antifungals, antiparasitics, or topical creams without notifying the researchers. Prior to week 10, dog #10, consuming FLX, withdrew from the study due to circumstances unrelated to the research trial or treatment diet.

**Table 1:** Mean age, mean body weight, breeds, and male:female and neutered:spayed:intact ratios of 30 client-owned dogs enrolled in a research trial investigating the effects of three oil supplements (camelina, canola, flaxseed) on transepidermal water loss, inflammatory and oxidative markers, and coat quality over a 16-week period.

**Treatment**	**Mean age (years)^a^**	**Mean BW (kg)^b^**	**Breeds**	**Male:female**	**Neutered:spayed:intact**
			Miniature dachshund		
			Havanese		
			Mix, unknown		
CAM	7.8	25	Mix, Australian shepherd/collie	2:8	2:7:1
			Mix, boxer whippet		
			Standard poodle		
			Norwegian elkhound		
			Labrador retriever (3)		
			Miniature dachshund		
			Pekingese		
			Mix, sled dog/unknown		
			Mix, border collie/sheltie		
FLX	7.7	27	Mix, husky/pointer	6:4	5:4:1
			Great dane		
			Standard poodle		
			Bernese		
			Labrador retriever (2)		
			Mix, mastiff/boxer		
			King Charles cavalier spaniel		
			Mix, samoyed/collie		
			Sheltie		
OLA	6.05	28	German shepherd	6:4	4:4:2
			Barbet		
			Standard poodle		
			Bernese		
			Labrador retriever (2)		

^a^Mean age of dogs on week 0 of research trial; units = years.

^b^Mean body weight of dogs on week 0 of research trial; units = kilograms.

BW, body weight; CAM, camelina oil; FLX, flaxseed oil; OLA, canola oil.

Treatment oils were provided at an inclusion level of 8.2 grams of oil per 100 grams total dietary intake.

### Dietary treatments

Over a 4-week wash-in period, all dogs were acclimated to a dry extruded commercial kibble (SUMMIT Three Meat Reduced Calorie Recipe, Petcurean, Chilliwack, BC, Canada; Table 2), sunflower oil (SA Kernel-Trade, Kuiv, Ukraine; Table 3), and beef-based treats (Beef Tendersticks, The Crump Group, Brampton, ON, Canada; proximate analysis: metabolizable energy 3039 kcal/kg; crude protein minimum 65%; crude fat minimum 5.1%; crude fiber maximum 4.0%; moisture max 9.56%). Oil was included in the diet at 8.2 grams of oil per 100 grams of total food intake, bringing the total dietary lipid content to 20% on an as-fed basis. Treats were included in the diet up to 2.5 grams per 100 grams total intake, and the remaining proportion of the diet was provided as kibble. During the wash-in period and throughout the study, daily portions of food, oil, and treats were pre-weighed by researchers and provided to the owners in 2-week intervals to be offered to dogs daily at a frequency determined by the owner. To avoid the occurrence of lipid peroxidation, owners were instructed to mix the oil with the food immediately before feeding. Any leftover kibble, oil, and/or treats were returned to researchers and subsequently weighed and recorded. Dogs were initially fed to meet their estimated maintenance energy requirements (110 kcal ME × kg BW^0.75^), and BW was recorded every 2 weeks starting at baseline. Each dog's food allotment was then adjusted accordingly to maintain baseline BW throughout the study. No abnormal observations were reported by owners throughout the 16-week study period in terms of diet tolerance (i.e., vomiting, stool quality, halitosis, etc.).

**Table 2:** Proximate analysis, metabolizable energy, omega-6 and omega-3, and linoleic and docosahexaenoic acid content of a commercial extruded kibble^a^ on an as-fed basis, fed to 30 client-owned dogs during a skin and coat health trial over a 16-week period.

**Nutrient profile**	**As fed basis**
Moisture (%)	8.00
Crude protein (%)	21.0
Nitrogen-free extract (%)	52.0
Crude fiber (%)	2.80
Crude fat (%)	9.00
Omega 6 (%)	2.00
Omega 3 (%)	0.20
Linoleic acid (%)	1.90
Docosahexaenoic acid (%)	0.01
Ash (%)	7.10
Metabolizable energy (kcal/kg)	3,324

^a^Chicken meal, whole brown rice, whole white rice, barley, oatmeal, chicken fat (preserved with mixed tocopherols), peas, lamb meal, salmon meal, natural chicken flavor, whole dried egg, sunflower oil, rice bran, flaxseed, dried kelp, dicalcium phosphate, potassium chloride, choline chloride, sodium chloride, calcium carbonate, vitamins (vitamin A supplement, vitamin D3 supplement, vitamin E supplement, niacin, L-ascorbyl-2- polyphosphate (a source of vitamin C), d-calcium pantothenate, thiamine mononitrate, beta-carotene, riboflavin, pyridoxine hydrochloride, folic acid, biotin, vitamin B12 supplement), minerals (zinc proteinate, iron proteinate, copper proteinate, zinc oxide, manganese proteinate, copper sulfate, ferrous sulfate, calcium iodate, manganous oxide, selenium yeast), DL-methionine, glucosamine hydrochloride, chondroitin sulfate, yeast extract, *Yucca schidigera* extract, dried rosemary.

**Table 3:** Analyzed fatty acid profiles of camelina oil, canola oil, flax oil, and sunflower oil fed to 30 client-owned dogs top dressed on commercial kibble during a skin and coat health trial over a 16-week feeding period.

**Parameter**	**Sunflower^a^**	**Canola^b^**	**Flaxseed^b^**	**Camelina^b^**
Saturated fatty acids (%)	9.61	6.50	8.20	9.50
Monounsaturated fatty acids (%)	14.1	63.8	16.6	35.2
Polyunsaturated fatty acids (%)	76.3	29.7	75.2	55.3
Omega 6 (%)	76.2	18.6	16.5	19.8
Omega 3 (%)	0.04	11.1	58.6	35.4

^a^Numerical values from Kostik et al. (25) and only represent generic sunflower oil, not the brand used for this study.

^b^Samples analyzed in duplicate by SGS Canada Inc. (Guelph, ON, Canada), average values reported.

Burron et al. (24).

### Study design

This study was conducted using a randomized complete block design (RCBD) with repeated measures. Following the 4-week wash-in period, dogs were blocked by breed, age, and BW and groups were randomly assigned to one of 3 treatment oils: camelina oil (CAM) (*n* = 10; eight females; two males), flaxseed oil (FLX) (*n* = 10; five females; five males), or canola oil (OLA) (*n* = 10; four females; six males). The sunflower oil used during the wash-in was replaced with either CAM, FLX, or OLA, and feeding continued as described for 16 weeks. Both OLA and FLX were chosen as control groups for this study as they are commonly used to formulate canine diets and provide a source of *n*-3 FAs.

### Blood collection

Dogs were fasted for a minimum of 10 h overnight and blood samples were collected *via* cephalic venipuncture using a syringe (Becton, Dickinson and Company, Franklin Lakes, NJ, USA). Of the collected blood, 5 mL was put into a serum vacutainer (Becton, Dickinson and Company, Franklin Lakes, NJ, USA). Blood was allowed to clot and was centrifuged at 7,200 × *g* for 15 min using an accuSpin Micro 17 centrifuge (Thermo Fisher Scientific, Waltham, MA, USA). Then, the serum aliquots were frozen at −80°C until later analysis.

### Inflammatory and oxidative markers

Serum samples were analyzed for prostaglandin E_2_ (PGE_2_) (Canine Prostaglandin E_2_ ELISA Kit MBS013017, MyBioSource, Vancouver, BC) and junction plakoglobin (JUP) (Canine Junction Plakoglobin ELISA Kit MBS104997, MyBioSource, Vancouver, BC) using commercially available ELISA (Enzyme-linked immunosorbent assay) kits. Samples were run in duplicate according to the manufacturer's instructions. Serum glycosaminoglycan (GAG) (dimethyl methylene blue) and nitric oxide (NO) (Griess Reaction; Molecular Probes, Eugene, OR) concentrations were determined using spectrophotometric assays (26, 27). Serum NO and GAG samples were analyzed as previously described by MacNicol et al. (28).

### Skin barrier function

Skin barrier function and integrity were assessed by measuring TEWL, which is defined as the amount of water that passively evaporates through skin to the external environment due to a water vapor pressure gradient on both sides of the skin barrier and is commonly used to characterize skin barrier function and integrity (29, 30). On weeks 0, 2, 4, 10, and 16, TEWL was measured using a VapoMeter^®^ SWL-3 (Delfin Technologies Ltd, Kuopio, Finland), according to the manufacturer's instructions. Since privately-owned dogs were used, it was not feasible to shave multiple patches for TEWL measurements, and as a result, researchers chose three body sites with little hair to measure TEWL, including: the right paw pad, right pinna, and right inner thigh. Ten measurements were taken per body site and the average was used for analyses. Once the averages were calculated, any values above or below the average by 50 g/m^2^/h or more were considered outliers and removed. All dogs were brought to the University of Guelph by their owners on collection days to ensure environmental conditions during collections remained consistent. All measurements were carried out by a single operator, in the same order of body sites, and in a climate-controlled room to maintain consistency between samples and to avoid variation in VapoMeter^®^ readings due to temperature and humidity fluctuations (29). Room conditions were stable at 22–23°C ambient temperature and 44–50% ambient relative humidity. The evaporation rate value is calculated in grams of water per square meter per hour (g/m^2^/h). All dogs were behaviorally acclimated to the use of the VapoMeter^®^, the researchers involved in sample collection, and the collection room, prior to the first sample day to minimize stress, thereby reducing variation in measurements. If dogs were wet due to weather upon arrival they were dried with a towel, to reduce variation further.

### Coat quality

Two researchers blinded to treatment were trained to perform a subjective coat assessment on weeks 0, 2, 4, 10, and 16 using a 5-point Likert scale (under Supplementary material). A Likert scale was used to measure the softness, shedding, dander, shine, spring, softness uniformity, color, color uniformity, and follicle density of the coat. Follicle density was assessed on the center of the back of the dogs by scoring the thickness/amount of hair coming from individual follicles. To increase consistency among dogs given different management practices in each household, all dogs were bathed 2 weeks prior to each assessment and owners were instructed to keep dogs dry and to not brush or groom them during this period.

### Statistical analysis

Data are presented as mean ± SD unless otherwise stated. All statistical analyses were performed using the PROC GLIMMIX of SAS Studio^®^ software (v.9.4., SAS Institute Inc., Cary, NC, USA). Dog was the experimental unit, and treatment, TEWL site, and sex, and age were treated as fixed effects (age and sex data not presented). Week was treated as a repeated measure. An analysis of variance (ANOVA) was performed to assess the effects of treatment on inflammatory and oxidative marker concentrations, TEWL, and coat scores. When the fixed effects were significant, the means were separated using Tukey–Kramer adjustments. Significance was declared at a *P* ≤ 0.05. Trends were declared at *P* ≤ 0.10.

## Results

### Inflammatory and oxidative markers

#### Prostaglandin E_2_

There were no differences among treatments (*P* = 0.973), across weeks (*P* = 0.397), or for treatment by week interactions (*P* = 0.987) (Table 4). Additionally, no differences were observed due to sex (*P* = 0.937) or age (*P* = 0.274).

**Table 4:** Serum prostaglandin E_2_, junction plakoglobin, glycosaminoglycan, and nitric oxide concentrations of healthy adult dogs supplemented one of three treatment oils^a^ on weeks 0, 2, 4, 10, and 16 of a skin and coat health trial, presented as lsmeans ± standard error.

	**Week**					***P*** **-values**		
	**0**	**2**	**4**	**10**	**16**	**Treatment**	**Week**	**Treatment** ^*^ **week**
**Prostaglandin E**_2_ **(pg/mL)**								
CAM	0.88 ± 1.45	2.77 ± 1.45	3.49 ± 1.45	2.35 ± 1.45	2.32 ± 2.33			
OLA	3.07 ± 1.34	3.07 ± 1.38	2.41 ± 1.44	2.82 ± 1.44	2.80 ± 1.39	0.9734	0.3965	0.9868
FLX	2.55 ± 1.23	4.07 ± 1.28	3.07 ± 1.34	3.44 ± 1.28	3.15 ± 1.33			
**Junction plakoglobin (ng/mL)**								
CAM	8.73 ± 1.08	9.38 ± 1.08	8.56 ± 1.11	8.65 ± 1.08	7.82 ± 1.08			
OLA	10.09 ± 1.01	9.60 ± 1.01	9.51 ± 1.01	9.96 ± 1.01	7.39 ± 1.09	0.9693	0.2487	0.9133
FLX	8.94 ± 0.94	10.97 ± 0.94	10.78 ± 0.94	9.34 ± 0.97	8.35 ± 1.02			
**Glycosaminoglycan (**μ**g/mL)**								
CAM	4.43 ± 0.73	4.73 ± 0.73	4.23 ± 0.73	4.91 ± 0.80	3.97 ± 0.76			
OLA	3.03 ± 0.73	4.34 ± 0.73	4.47 ± 0.72	4.17 ± 0.76	3.74 ± 0.72	0.2083	0.9945	0.9147
FLX	4.33 ± 0.66	4.50 ± 0.66	4.82 ± 0.69	4.85 ± 0.69	4.04 ± 0.78			
**Nitric oxide (**μ**M/mL)**								
CAM	2.20 ± 5.50	9.30 ± 5.50	4.82 ± 5.62	8.34 ± 5.60	10.90 ± 5.64			
OLA	4.31 ± 5.05	7.19 ± 5.05	5.85 ± 5.05	9.26 ± 5.05	10.15 ± 5.18	0.6476	0.3587	0.7288
FLX	11.70 ± 4.58	12.76 ± 4.58	19.56 ± 4.72	13.74 ± 4.72	16.34 ± 4.72			

^a^Treatment oils: CAM, Camelina; OLA, Canola; FLX, Flaxseed oil; Data presented as mean ± standard error; *n* for each treatment group on weeks 0, 2, 4: CAM = 10, OLA = 10, FLX = 10, and weeks 10, 16: CAM = 10, OLA = 10, FLX = 9.

#### Junction plakoglobin

There were no differences among treatments (*P* = 0.969), across weeks (*P* = 0.249), or for treatment by week interactions (*P* = 0.913) (Table 4). No differences were observed due to sex (*P* = 0.914) or age (*P* = 0.743).

#### Glycosaminoglycan

There were no differences among treatments (*P* = 0.208), across weeks (*P* = 0.995), or for treatment by week interactions (*P* = 0.915) (Table 4). Concentrations of GAG tended to be greater in males compared to females (*P* = 0.078). There were no differences observed due to age (*P* = 0.329).

#### Nitric oxide

There were no differences among treatments (*P* = 0.648), across weeks (*P* = 0.359), or for treatment by week interactions (*P* = 0.729) (Table 4). No differences were observed due to sex (*P* = 0.226) or age (*P* = 0.424).

### Transepidermal water loss

Of the 4,440 individual TEWL measurements collected throughout the study period, 18 were considered outliers and removed [D = Dog, W = Week; Paw pad: D6W2(CAM), D8W16(FLX)(2 values), D9W16(FLX), D17W4(CAM), D18W2(FLX), D18W4(FLX)(2 values), D23W10(CAM), D23W16(CAM); Inner ear: D5W4(OLA), D5W10(OLA), D12W10(OLA); Inner leg: D6W2(CAM), D6W10(CAM), D12W0(OLA), D16W0(FLX), D29W0(FLX)]. These outliers could often be attributed to changes in the environment, leading to signs of stress or excitement in the dogs (i.e., researchers entering and leaving the room, noises occurring outside of the sample room, and in the case of some outliers these samples were taken near the end of the collection period and the dogs would become impatient, no longer wanting to remain in the same spot for samples).

There were no differences among treatments (*P* = 0.726), across weeks (*P* = 0.738), or for treatment by week interactions (*P* = 0.996). Additionally, there were no differences for site by week (*P* = 0.378), or sex (*P* = 0.274) (Table 5). However, there were differences observed among sites (*P* < 0.0001), in that TEWL values for the paw pad were greater than those of the pinna or inner thigh. Additionally, there was a trend observed in age (*P* = 0.072), in that senior dogs (11–14 years; *n* = 3) tended to have lower mean TEWL values compared to young (2–4 years; *n* = 7), young adult (5–7 years; *n* = 9), and adult dogs (8–10 years; *n* = 9).

**Table 5:** Mean transepidermal water loss (TEWL) values (g/m^2^/h) of the right paw pad, right pinna, and right inner thigh of healthy adult dogs supplemented one of three treatment oils^a^ on weeks 0, 2, 4, 10, and 16 of a skin and coat health trial, presented as lsmeans ± standard error.

		**Week**					***P*** **-values**		
**Treatment**	**Site**	**0**	**2**	**4**	**10**	**16**	**Trt**	**Site**	**Week**
CAM	Paw pad	92.57 ± 8.80	98.97 ± 8.80	88.28 ± 8.80	83.98 ± 8.80	92.7 ± 8.80			
OLA	Paw pad	88.27 ± 8.85	86.95 ± 8.85	76.32 ± 8.85	71.38 ± 8.85	67.56 ± 8.85			
FLAX	Paw pad	99.43 ± 8.79	109.51 ± 8.79	100.37 ± 8.79	87.38 ± 9.21	88.46 ± 9.21			
CAM	Pinna	14.03 ± 8.80	12.27 ± 8.80	18.78 ± 8.80	14.47 ± 8.80	16.68 ± 8.80			
OLA	Pinna	14.43 ± 8.85	15.84 ± 8.85	16.87 ± 8.85	24.43 ± 8.85	18.40 ± 8.85	0.7261	< 0.0001	0.7375
FLAX	Pinna	9.10 ± 8.79	12.69 ± 8.79	12.13 ± 8.79	13.27 ± 9.21	9.92 ± 9.21			
CAM	Inner thigh	23.11 ± 8.80	23.56 ± 8.80	18.2 ± 8.80	17.52 ± 8.80	22.93 ± 8.80			
OLA	Inner thigh	16.86 ± 8.85	15.72 ± 8.85	18.18 ± 8.85	17.32 ± 8.85	21.23 ± 8.85			
FLAX	Inner thigh	15.7 ± 8.79	13.44 ± 8.79	16.36 ± 8.79	14.30 ± 9.21	16.51 ± 9.21			

^a^Treatment oils: CAM, Camelina; OLA, Canola; FLX, Flaxseed oil; Data presented as mean ± standard error; *n* for each treatment group on weeks 0, 2, 4: CAM = 10, OLA = 10, FLX = 10, and weeks 10, 16: CAM = 10, OLA = 10, FLX = 9.

### Coat quality

#### Softness

**Figure 1:**
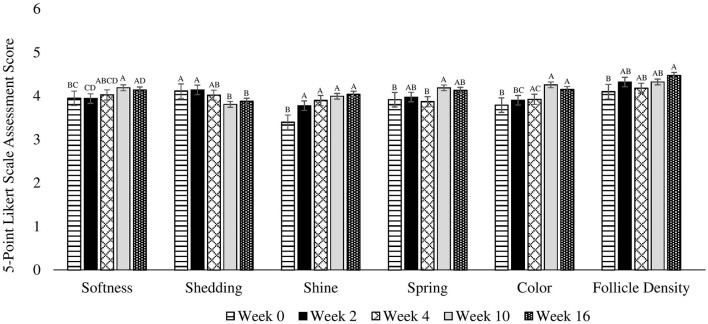
Mean coat quality assessment scores completed using a 5-point Likert scale on 30 client owned healthy adult dogs fed one of three treatment oils (camelina oil, canola oil, flaxseed oil) and commercial kibble. ^A, B, C, D^Bars without a common letter differ significantly (*P* < 0.05).

There were no differences among treatments (*P* = 0.539), for treatment by week interactions (*P* = 0.757), or due to age (*P* = 0.479), week by age (0.338) or week by sex (*P* = 0.738) interactions. However, there were differences observed across weeks for pooled data (*P* = 0.005) in that softness was greater on week 10 and 16 compared to week 0, and greater on week 10 compared to week 2. Week 4 was not different from any other time points (Figure 1). Additionally, softness was greater in females compared to males (*P* = 0.026).

#### Shedding

There were no differences among treatments (*P* = 0.882), due to age (0.894) or sex (*P* = 0.760), or for treatment by week (*P* = 0.444), week by age (*P* = 0.302), or week by sex (*P* = 0.514) interactions. For pooled data across weeks, shedding was greater on weeks 0 and 2 compared to weeks 10 and 16 (*P* = 0.004). Week 4 was not different from any other time points (Figure 1).

#### Dander

There were no differences among treatments (*P* = 0.648), due to age (*P* = 0.114) or sex (*P* = 0.349), across weeks (*P* = 0.129), or for treatment by week (*P* = 0.869), week by age (*P* = 0.171), or week by sex (*P* = 0.163) interactions (Figure 1).

#### Shine

There were no differences among treatments (*P* = 0.815), due to age (*P* = 0.945), or sex (*P* = 0.191), or treatment by week (*P* = 0.998), week by age (0.992), or week by sex (*P* = 0.375) interactions. However, there were differences across weeks for pooled data (*P* < 0.0001) in that shine on weeks 2, 4, 10, and 16 was greater than at week 0 (Figure 1).

#### Spring

There were no differences among treatments (*P* = 0.918), due to age (*P* = 0.663) or sex (*P* = 0.401), or for treatment by week (*P* = 0.397), week by age (*P* = 0.773), or week by sex (*P* = 0.997) interactions. However, there were differences across weeks for pooled data (*P* = 0.014) in that spring was greater on week 10 compared to week 4 and 0. There were no differences on weeks 2 and 16 (Figure 1).

#### Softness uniformity

There were no differences among treatments (*P* = 0.969), due to age (*P* = 0.860) or sex (*P* = 0.132), or for treatment by week (*P* = 0.799), week by age (*P* = 0.996), or week by sex (*P* = 0.142) interactions. However, a trend was observed across weeks for pooled data (*P* = 0.065) in that softness uniformity tended to be greater on week 16 compared to week 0. Weeks 2, 4, and 10 were not different from any other time points (Figure 1).

#### Fur color

There were no differences among treatments (*P* = 0.323), due to age (*P* = 0.770) or sex (*P* = 0.546), or for treatment by week (*P* = 0.567), week by age (*P* = 0.345), or week by sex (*P* = 0.954) interactions. However, there were differences across weeks for pooled data (*P* < 0.0001) in that color was higher on weeks 4, 10, and 16 compared to week 0. Additionally, color was greater on week 10 and 16 compared to week 2. Furthermore, color tended to be higher on week 10 compared to week 4 (Figure 1).

#### Fur color uniformity

There were no differences among treatments (*P* = 0.541), due to age (*P* = 0.893) or sex (*P* = 0.911), across weeks (*P* = 0.362), or for treatment by week (*P* = 0.291), week by age (*P* = 0.787), or week by sex (*P* = 0.910) interactions (Figure 1).

#### Follicle density

There were no differences among treatments (*P* = 0.873), due to age (*P* = 0.795) or sex (*P* = 0.854), or for treatment by week (*P* = 0.670), week by age (*P* = 0.846), or week by sex (*P* = 0.299) interactions. However, there were differences across weeks for pooled data (*P* = 0.027) in that follicle density was greater on week 16 compared to week 0. Weeks 2, 4, and 10 were not different from any other time points (Figure 1).

## Discussion

The purpose of this study was to assess the effects of camelina oil supplementation on skin and coat health compared to canola and flaxseed oil, two oils currently used to formulate canine diets. The results presented herein suggest no differences in TEWL, coat quality, or the inflammatory and oxidative markers assessed due to treatment over the 16-week period.

### Inflammatory and oxidative markers

In the current study, concentrations of GAG tended to be higher in males compared to females. Studies in humans by (1) Larking (31) and (2) Claassen and Werner (32) found that, similar to the present study, females have lower concentrations of GAG. Claassen and Werner analyzed GAG in thyroid cartilage while Larking measured GAG excretion in the tissue. Since GAG is a marker of cartilage turnover, Claassen and Werner attribute their findings to greater cartilage turnover in males, while Larking accredits their findings to the males in their study having a greater mean height (31, 32). It is possible that the female dogs in the present experiment had a smaller average height and lower cartilage mineralization than the males, which contributed to the lower concentration of circulating GAGs observed. However, height and cartilage mineralization were not measured in the present study. Furthermore, the observation made in our study was only a tendency; this, combined with the dearth of work carried out in dogs and lack of equal distribution of male/female, intact/neutered/spayed dogs in the current study make it difficult to form any cogent conclusions. Future research should investigate this relationship further using a dog model.

No significant changes were observed in PGE_2_, JUP, GAG, or NO concentrations over the 16-week study period. It is possible that the stability of these concentrations across time and among treatments is attributed to the lack of exercise or immune challenge experienced by the dogs on the current study. It is well-established that both exercise and immune challenges result in a wide range of physiological and biochemical adaptations, the magnitude of which is directly related to the intensity and duration of the exercise or immune challenge encountered (33–36). This wide range of physiological and biochemical adaptations include changes in inflammatory and oxidative biomarker concentrations (28, 33).

Dogs and horses both experience increased PGE_2_ concentrations following exercise. In horses, NO and GAG concentrations increase following exercise and compared to baseline, but no change was observed in dogs (28, 33). Pearson et al. attribute these results, similar to previous findings, to variations in NO production depending on exercise intensity, suggesting that it is possible that the lack of changes observed in NO concentration in the current study is due to the low intensity of the exercise experienced by the dogs (33). Markers like PGE_2_, NO, GAG, and JUP are often upregulated during times of immune challenge/disease (37–40). A myriad of studies completed in humans suggest no effects of *n*-3 PUFA supplementation on inflammatory or immune markers in healthy individuals (41–43). As an example, Pot et al. found that supplementing fish oil and sunflower oil to healthy individuals had no effect on chemokine, cytokine, or cell adhesion molecule concentration compared to baseline (41). Healthy individuals, similar to the canine subjects of our study, generally have low levels of circulating inflammatory markers. Thus, the chance that low levels of inflammation are reduced even further by an intervention with oil is very small and difficult to measure. The dogs of the present study were healthy upon recruitment and on every sample period based on a veterinary examination, as well as CBC and biochemistry analysis, indicating a lack of immune response that would elicit an inflammatory response. Additionally, the dogs did not participate in any intense exercise prior to or on sample days, and thus had no known reason to elicit any exercise stress induced response impacting markers of inflammatory or oxidative stress. For safety and animal care purposes, no procedures with the potential to cause harm to the animals, like an inflammatory or immune challenge, can be carried out in client-owned dogs. Additionally, the objective of the present study was to determine how these three oils compare to one another in terms of their effects on these biomarkers to gauge their use in dog food formulations for typical pets, not to evaluate their performance following an exercise or immune challenge. Future studies should compare the effects of these three oils and their performance following exercise and immune challenge.

### Transepidermal water loss

In the present study, mean TEWL values were significantly greater when measured on the paw pad compared to the inner leg and inner ear. This is likely the result of the tubular, unbranched eccrine glands that open directly onto the skin of the paw pads and noses of canines. These glands allow sweat to be released from these areas, contributing to the water-loss detected by the VapoMeter, and thereby likely contributing to greater TEWL values compared to the inner leg and pinna (44). Additionally, TEWL values were found to be lower in senior dogs compared to young, young adult, and adult dogs. Similar findings have been observed in other canine and human studies and although the exact mechanism behind these observations is unclear, there are various theories (45, 46). The thickness of the stratum corneum and flattening of corneocytes increases with age, while natural moisturizing factors, stratum corneum hydration, and epidermal lipid synthesis are reduced (47–53). Additionally, the density of dermal capillaries decreases with age, which may lower skin temperature and in turn decrease water diffusion (51, 54). All of these findings provide examples of mechanisms that increase the path length and resistance of a water molecule and subsequently contribute to lower TEWL in older individuals, and in agreement with the present study.

### Coat quality

Spring and follicle density increased significantly from baseline. This is likely due, at least in part, to the growth of winter coats as the study began at the end of summer and went into the winter (September–January). Dogs have a light summer undercoat that is shed before a thick winter undercoat grows in, which could explain the increase in spring and follicle density. This further supports the observation of the present study in that shedding was greater in all dogs at the beginning of the study at weeks 0 and 2, compared to weeks 10 and 16.

Softness, shine, and color of the dogs' coats increased from baseline. This is likely a result of the dogs consuming an increased amount of *n*-3 FAs following baseline, which can be further metabolized into EPA and DHA, though with limited efficiency. Supplementation of fish oil, a rich source of EPA and DHA, was found to improve skin and hair coat quality in dogs from baseline based on a clinical score, with maximal improvement occurring after 8 weeks (55). The positive effects on skin and coat health are thought to be due to an increase in EPA and DHA in the erythrocyte membrane, along with increased total lipids in the hair shaft (55). The same study observed that following supplement withdrawal, skin and coat health clinical scores remained the same for 1 month and began to deteriorate following the second month (55). Although we did not take measurements on week 8, we did take measurements on week 10, and this is where we saw the largest improvement (i.e., softness, shedding, shine, spring, and color). This is most likely due to the increase in ALA, which is the parent compound of EPA and DHA, the dogs received from their treatment oil (CAM 1:1.8, FLX 1:4.19, OLA 1:0.59) in comparison to the wash-in sunflower oil (1:0). It is important to note that our study had no negative control group, since the absence of an oil supplement would alter all macronutrient intakes and our aim was to compare to existing approved oil supplements. As a result it cannot be ruled out that the observed changes in coat quality may be a result of the placebo effect. Future studies should consider employing a control group fed no oil supplement to rule out the possibility of the placebo effect impacting observations.

All dogs in the current study were considered healthy, with no known dermatological conditions or skin disorders. The coats of these dogs were in relatively good condition at baseline, and future research should investigate these oil supplements and their effects on skin and coat health in dogs with poor skin and coat quality as a result of conditions like atopic dermatitis. It is important to note that ectoparasites, particularly fleas in dogs, can negatively impact skin and coat health (56). In this study, although complete blood count and biochemistry values were assessed, and physical examinations were performed by a licensed veterinarian prior to study recruitment and throughout the entire trial, diagnostic and preventive control in terms of ectoparasites was not considered, and this is a limitation of this study. Authors recommend future studies consider using more specific techniques as inclusion criteria when recruiting participants in order to ensure the absence and prevention of parasites and their potential impact on skin and coat health.

## Conclusion

In conclusion, camelina oil is comparable to canola and flaxseed oil in terms of its effects on skin barrier function, coat quality, and the circulating inflammatory and oxidative markers measured in the current study when fed to healthy adult dogs, subjected to no physical or immunological challenge, and observed for 16-weeks. Canola and flaxseed oil are commonly used in canine food formulations. Flaxseed oil specifically has the ability to support skin and coat health claims, making camelina oil a potential alternative plant-based oil source with high concentrations of ALA that could contribute to achieving the ideal *n*-6:*n*-3 ratio in canine diets, while supporting skin and coat health claims.

## Data availability statement

The raw data supporting the conclusions of this article will be made available by the authors, without undue reservation.

## Ethics statement

The animal study was reviewed and approved by University of Guelph Animal Care Committee. Written informed consent was obtained from the owners for the participation of their animals in this study.

## Author contributions

AS and WP: conceptualization and funding acquisition. AS, WP, and DM: methodology. TR, SB, KP, and CG: study conduct. TR: formal analysis and writing—original draft preparation. TR, SB, DWM, CG, KP, LT, DM, WP, and AS: writing—reviewing and editing. All authors have read and agreed to the published version of the manuscript. All authors contributed to the article and approved the submitted version.
