# Supplementary material for: Effects of dietary camelina, flaxseed, and canola oil supplementation on inflammatory and oxidative markers, transepidermal water loss, and coat quality in healthy adult dogs
Source: Front Vet Sci. 2023 Mar 9;10:1085890. doi: 10.3389/fvets.2023.1085890 (PMC10034026; doi:10.3389/fvets.2023.1085890)
Supplement: Supplementary file 1 [file Data_Sheet_1.PDF]

Coat Quality Subjective Assessment

Evaluator Name:

Dog Name:

Date:

Coat assessment

	Score	1	2	3	4	5
Shedding - one pass		Severe loss	Excessive loss	Moderate loss	Minimal loss	No hair loss
Dander - back		Severe	Excess	Medium	Slight	None
Shine		Dull	Poorly reflective	Slightly reflective	Somewhat shiny	Glistens
Spring		Resistant	Slightly resistant	Bouncy	Partial return	Spontaneous
Softness		Extremely coarse	Moderately coarse	Acceptable feel	Moderately soft	Extremely soft
Softness uniformity		Extremely non-uniform in softness	Not very uniform	Acceptably uniform	Moderately uniform	Extremely uniform
Color		Extremely dingy	Slightly dingy	Typical	Slightly intense	Extremely intense
Color uniformity		Extremely non-uniform in color	Not very uniform	Acceptably uniform	Moderately uniform	Extremely uniform
Follicle density		Extremely thin	Fairly thin	Slightly dense	Fairly dense	Extremely dense

Notes

Supplementary material: A 5-point Likert scale used to assess coat quality in 30 client-owned dogs enrolled in a research trial investigating the effects of three oil supplements (camelina, canola, flaxseed) on transepidermal water loss, inflammatory and oxidative markers, and coat quality over a 16-week period.
